# Treating prolonged grief disorder with CBT for insomnia: A replicated single-case experimental study protocol

**DOI:** 10.1371/journal.pone.0341802

**Published:** 2026-02-12

**Authors:** Thomas A. de Lang, Peter J. de Jong, Marike Lancel, Jaap Lancee, Maarten C. Eisma

**Affiliations:** 1 University of Groningen, Groningen, the Netherlands; 2 GGZ Drenthe Mental Institute, Centre of Expertise on Sleep and Psychiatry, Assen, the Netherlands; 3 University of Amsterdam, Amsterdam, the Netherlands; Public Library of Science, UNITED STATES OF AMERICA

## Abstract

The most effective treatments of prolonged grief disorder (PGD) result in clinically relevant effects in only about half of the patients and can be emotionally taxing. This points to the importance of improving currently available treatment options. One promising target for enhancing the efficacy of the treatment of PGD is insomnia. Sleep disturbances are very common in bereavement and are proposed to play a causal role in maintaining PGD symptoms. Therefore, targeting sleep problems may be an effective treatment for people with PGD and insomnia disorder. This protocol presents a study, registered in the Dutch Trial Register (NL86238.042.24) that will evaluate the effects of cognitive behavioral therapy for insomnia (CBT-I) in individuals with comorbid PGD and insomnia using a replicated single-case experimental design (R-SCED). Twenty adults meeting diagnostic criteria for both disorders will be randomized to complete baseline between 5 and 14 weeks, after which they will receive CBT-I. Weekly PGD and insomnia symptom measures will be administered throughout the baseline (5–14 weeks), intervention (7–8 weeks) and post-intervention phase (4–13 weeks). Outcomes will be examined using visual inspection, Tau-U indices, and randomization tests. Results: We expect CBT-I to reduce both insomnia and PGD symptoms. This study will form the first systematic evaluation of CBT-I for PGD. Findings may help establish a novel, less emotionally demanding treatment option for bereaved individuals.

## Introduction

The death of a loved one is one of the most distressing experiences that people will face during their lifetime. While most individuals manage to cope with the loss using their own coping strategies, a small but notable subset of bereaved adults experience persistent, severe, and disabling grief, also termed prolonged grief (3–4%; [1,2]). Prolonged grief disorder (PGD) has recently been introduced in the Diagnostic and Statistical Manual of Mental Disorders (5th edition, text revision, DSM-5-TR; [3]) and the International Classification of Diseases-11^th^ edition (ICD-11; [4]). Core symptoms include persistent and severe yearning and/or cognitive preoccupation with the deceased, combined with secondary symptoms indicative of emotional pain, experienced for a prolonged period following bereavement (a minimum of six months for the ICD-11 and one year for the DSM-5-TR).

Recent systematic reviews and meta-analyses indicate that the most effective and well-studied prolonged grief-tailored interventions, cognitive behavioral therapy (CBT), show medium to large effect sizes [5,6]. However, clinically relevant changes are observed in no more than half of the participants [7,8], leaving many treatment-seeking bereaved people underserved. Additionally, CBT for prolonged grief can be emotionally taxing for patients as a key component is exposure to the reality of the loss [9] and drop-out rates are relatively high (e.g., [10]). Thus, expanding the treatment options for people dealing with severe and persistent grief reactions is important.

One potential target for the treatment of prolonged grief is sleep disturbances. Sleep disturbances are among the most common complaints of the bereaved [11,12], and it has been argued that sleep problems play a causal role in the development and maintenance of prolonged grief symptoms [13]. A systematic review showed that sleep problems are often comorbid with prolonged grief, the severity of insomnia symptoms is related to the severity of the prolonged grief symptoms both concurrently and longitudinally, and sleep problems do not fully remit after psychotherapy for PGD [13].

Recent longitudinal studies yield evidence consistent with the view that sleep disturbances contribute to the perpetuation of prolonged grief symptoms. For example, a study investigating the relation between spontaneous fluctuations in insomnia and prolonged grief symptoms and vice versa over the course of a year, showed that fluctuations in insomnia symptoms were predictive of changes in prolonged grief symptoms six months later [14]. Additionally, a large Dutch cohort study showed that sleep problems prior to bereavement, were predictive of experiencing prolonged grief at follow-up (median of six years later; [15]. Similarly, another longitudinal study [16] linked the typical trajectories of insomnia symptoms following a loss with the chance of experiencing PGD“caseness” at the one-year follow-up. Bereaved adults who showed continuous clinical insomnia symptom levels were more likely to have probable PGD at one-year follow-up compared to participants showing recovery, characterized by reduced insomnia symptom levels over time, or showed resilience, characterized by non-clinical symptom trajectories. Interestingly, the trajectories of insomnia symptoms and prolonged grief symptoms were strongly correlated, suggesting that these symptoms are intertwined and move together over time [16]. Together, these findings are consistent with the view that sleep problems constitute a risk factor for prolonged grief, and that targeting sleep disturbances in therapy may be an effective approach for people with comorbid PGD and insomnia disorder.

The exact mechanisms by which sleep might affect mental health problems is unclear. However, it has been hypothesized that sleep, especially rapid eye movement sleep (REM-sleep), plays a crucial role in detangling the emotional response from an emotional memory while simultaneously encoding the memory in the autobiographical memory base [17,18]. Insomnia is typically associated with fragmented (‘restless’) REM-sleep, which may disrupt this overnight adaptation to experienced negative emotions and distress (e.g., [19,20]). Based on these theoretical notions and empirical findings, we suggest that targeting insomnia (i.e., long-lasting (≥ 3 months) and frequent (≥ 3 nights a week) problems with falling and/or staying asleep, causing daytime distress/impairments) (DSM-5-TR; [3]), could be a viable new treatment option for people with comorbid PGD and insomnia disorder.

The effect of insomnia therapy in people with PGD has thus far not been systematically investigated. One small pilot study, investigated the effect of CBT-I on prolonged grief symptoms [21]. In this study, parents bereaved of a child by cancer, who reported clinically relevant insomnia symptoms, received CBT-I. Besides reductions in sleep disturbances, they reported large within-group reductions (*d* = 1.0) on prolonged grief symptomatology at the 9-month follow-up and even larger reductions at the 18-month follow-up (*d* = 2.6). However, the sample size of the study was small and between-group effects were not significant. Eight participants received CBT-I, which were compared with eight active control participants receiving information on sleep hygiene, stress management, and mindfulness. Although this study provides a valuable preliminary indication that targeting insomnia symptoms might be beneficial in the treatment of PGD, the choice of design, the limited number of treatment-receiving participants, and the specific sample do not allow any final conclusions with regard to the efficacy of this approach. Therefore, the current study was developed to further investigate the potential effect of CBT-I in people with PGD and insomnia disorder in a well-powered study.

The problem of small sample sizes in treatment studies is common in the field of bereavement studies (cf. [5,8]). When working with small samples, randomized controlled trials are often problematic. The assumptions required for traditional parametric model-based inference are often violated, and power issues arise. The current study aims to overcome these difficulties by employing a replicated single-case experimental design (R-SCED).

Unlike traditional group designs such as randomized controlled trials, R-SCED relies on design-based inference, which does not depend on assumptions about data distributions. Instead, assumptions are made about the design of the study. Furthermore, the R-SCED relies on within-subject comparisons; in other words, each participant serves as their own control. Combined with randomized baseline lengths, frequent measurements both during the baseline and intervention phases, as well as replication across multiple individuals, this allows for causal inference with a limited number of participants [22,23]. Based on the theoretical notions and empirical evidence outlined above, we hypothesized that targeting insomnia with the gold standard, evidence-based CBT-I (e.g., [24,25]), in people dealing with comorbid PGD and insomnia disorder, will reduce both prolonged grief and insomnia symptom levels.

## Method

### Participants

Dutch-speaking adults (> 18), who are bereaved of a loved one and meet the diagnostic criteria for insomnia disorder and PGD per DSM-5-TR are eligible for participation in the study. Exclusion criteria are other untreated sleeping disorders, recent shift in sleep medication (< 3months), shift work, traveling between time zones, being diagnosed with a psychotic disorder or having concrete suicidal ideations. Participants will receive financial compensation for each completed questionnaire with a maximum compensation of 180 euro.

### Procedure

The study was approved by the Medical Ethical Committee at the University Medical Center Groningen (UMCG) in the Netherlands (METc 2024/422) and was registered in the Netherlands Trial Register (NL86238.042.24) (https://www.onderzoekmetmensen.nl/en/trial/57646). Participants will be recruited through ongoing research, localized Google AdWords advertisements, and websites for organizations for bereaved persons, linking to a research website. On the website, interested bereaved adults can access information about the study and can provide informed consent on a secure online survey program, Qualtrics. After providing informed consent, participants proceed to an online screener to preliminarily check for the inclusion and exclusion criteria. The screener includes questionnaires on sociodemographic and loss-related variables, prolonged grief symptoms, insomnia and other sleep disorder symptoms (see Materials).

People who meet the established cut-off values for insomnia disorder (a score above 14 on the Insomnia Severity Index; ISI) and PGD (a score of 33 or higher on the DSM-5-TR items of the Traumatic Grief Inventory Self-Report Plus; TGI-SR + ; [26]) based on the screener will be invited to meet online (via a secure online meeting program) or to visit the research institute for a clinical interview to assess PGD (Traumatic Grief Inventory-Clinician Administered, TGI-CA; [27]) and insomnia disorder (Structured Clinical Interview for DSM-5 Sleep Disorders, SCISD; [28]). When participants meet the criteria for PGD, insomnia disorder and none of the exclusion criteria, they can immediately start the waiting period for CBT-I. The duration of the waiting period will be randomly assigned and lasts between five and fourteen weeks. A random number between 5 and 14 (resembling the waiting time) will be drawn using randomizer.org for each participant after the clinical interview has been conducted.

**Fig 1:**
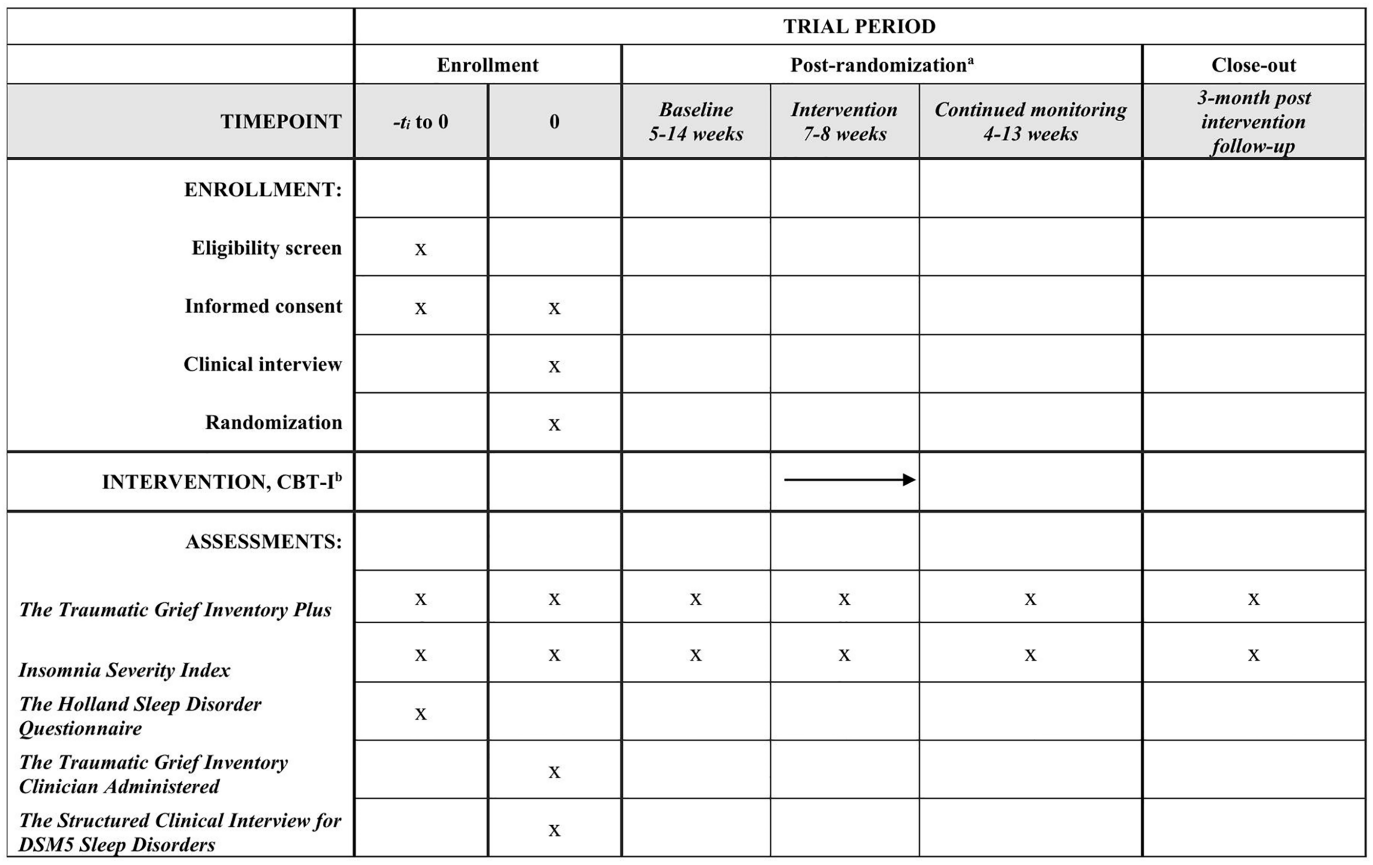
Schedule of enrollment, interventions, and assessments. ^a^ Assessments will be conducted weekly (26 weeks in total). ^b^ Cognitive behavioural therapy for insomnia.

Once the waiting period has started, participants will receive weekly questionnaires assessing prolonged grief symptoms (TGI-SR+) and insomnia symptoms (ISI). These weekly questionnaires will be administered for 26 weeks, covering the waiting period (between 5–12 weeks), intervention phase (7–8 weeks), and a post-intervention/continued monitoring phase (between 4–13 weeks). Following the waiting period, all participants will receive CBT-I following the standard Dutch protocol [29] by a trained psychologist, supervised by a licensed healthcare psychologist. The therapy can be delivered in person and online, depending on the location and preferences of the participant. Three months post-treatment, participants will be asked to complete the ISI and TGI-SR+ once more (see Fig 1). We expect to start data collection in March 2026, to complete data collection in June 2027 and, report our results in October 2027.

Participants who still experience severe and disabling grief reactions (TGI-SR + score > 33, on the DSM5-TR items; 26) at the 3-month follow-up will be offered the possibility of CBT for PGD delivered by the same psychologist. CBT for PGD is the gold standard treatment for PGD and can be effectively delivered in person and online [5,30].

### Materials

#### Questionnaires.

Socio-demographic (age, sex, and education level) and loss-related characteristics (kinship to the deceased, expectedness of the loss, time since loss, and cause of death) will be assessed with a self-constructed questionnaire, included in the screener.

Prolonged grief symptoms will be assessed with the Traumatic Grief Inventory Self-Report Plus (TGI-SR + ; [26]). This 22-item questionnaire is based on the TGI-SR [31] and was developed to measure all symptoms associated with the different criteria sets of grief disorders as included in the DSM-5 (persistent complex bereavement disorder), DSM-5-TR and ICD-11 (PGD). In the current study, the 12 items concerning DSM-5-TR symptoms will be used. Participants will be asked to indicate to what extent symptoms were experienced during the last month for the screener and during the last week for the repeated weekly measurement. Items are scored on a Likert scale from 1 (never) to 5 (always). Higher sum scores represent higher prolonged grief symptom levels. Total sum scores of 33 or higher are indicative of probable PGD [26].

The Dutch translation of the Insomnia Severity Index (ISI; [32,33]) will be used to assess insomnia symptoms. The seven items contain questions on nighttime and daytime symptoms and worries about sleep. Participants are asked to rate the severity of the symptoms during the last 2 weeks for the screener and during the last week for the repeated weekly measurement. Items are scored on a 5-point Likert scale from 0 to 4 (varying anchors). Higher sum scores represent more severe insomnia symptoms. Total scores range from 0 to 28. Scores of 8–14 are considered to indicate subthreshold insomnia and scores above 14 are indicative of clinical insomnia [32].

The Holland Sleep Disorder Questionnaire (HSDQ; [34]) is a validated, Dutch, self-assessment questionnaire to screen for sleep disorders, such as insomnia disorder, sleep-related breathing disorders, and sleep-related movement disorders. The HSDQ will be used in the screener to assess for the presence of other sleep disorders.

#### Clinical interviews.

To assess the inclusion and exclusion criteria; the presence of both PGD and insomnia disorder as well as ruling out the presence of other sleep disorders, we plan to administer two semi-structured clinical interviews.

The Traumatic Grief Inventory Clinician Administered (TGI-CA; [27]) will be used to assess the presence of PGD per DSM-5TR criteria. This semi-structured interview was developed to assess all current criteria for disturbed grief, including PGD per ICD-11 and DSM-5-TR. The interview questions related to the DSM-5-TR criteria will be used in the current study.

The Structured Clinical Interview for DSM-5 Sleep Disorders (SCISD; [28]) will be used to assed the presence of insomnia disorder and to rule out the presence of other sleep disorders. The SCISD is a well-validated clinical interview to assess all DSM-5 sleep disorders, including insomnia disorder.

### Treatment

The CBT-I protocol consists of six to seven weekly sessions with a booster session at one-month follow-up [29]. Participants will receive a take-home textbook during the first session which they can use to follow along with the therapy sessions.

The first two sessions focus on psycho-education, through providing information about the function and regulation of normal sleep and the consequences of disturbed sleep, sleep hygiene, a healthy lifestyle that can improve sleep and, behaviours that disrupt sleep. During session three, stimulus control and sleep restriction will be introduced. Stimulus control strengthens the association between bed (and bedroom) and sleep by only going to bed when sleepy and leaving the bed when awake for longer than 30 minutes and getting up at a fixed time. Sleep restriction restricts bedtime to the average subjective total sleep duration during the past week (≥ 5 hours), thereby elevating sleep pressure, which will result in a higher relative percentage of time slept while in bed (sleep efficiency). Based on reported sleep efficiency (SE) during the past week and the participant’s age, bedtimes will be extended or reduced (see Table 1). Sessions 4–6 focus on cognitive therapy, identifying and challenging misconceptions and worries about sleep that keep a person awake, and on relaxation, employing various relaxation techniques to teach a person to unwind. The final session focuses on relapse prevention. Four weeks after the final session, a booster session is offered in which key elements of the treatment will be revisited if needed.

**Table 1:** Rules for extending and reducing time in bed.

Age	Extend by 15 min	Reduce to average sleep time
< 50	> 85%	< 85%
> 50	> 80%	< 80%
> 70	> 70%	< 70%

### Sample size

Based on a prior small pilot study on CBT-I effects on prolonged grief symptoms [21] and studies on the effect of CBT-I on depression symptoms [35,36], we anticipate a large effect size (Cohen’s *d* = 1.0) on prolonged grief and insomnia symptoms. An a priori power analysis was conducted using the single case designs shiny web-app for power calculations [37]. Based on power of.80, to detect a large effect, a total of 26 measurements per participant, of which minimally five during baseline phase, followed by minimally 12 measurements during the treatment (and continued monitoring) phase and an alpha of.05, sample size of 19 is required. A total of 20 patients will be enrolled to ensure sufficient power. CBT-I generally elicits a low dropout rate (e.g., [38]); if participants drop out before the fourth session, other participants will be recruited to replace them.

### Analysis

To test the hypothesis that prolonged grief symptoms will decline during the treatment of comorbid insomnia we will use two analytic strategies (cf. [39]). First, we will use visual inspection. The data of each participant will be plotted separately. We will then inspect changes in the mean level of prolonged grief symptoms between the baseline phase and the treatment phase. The mean level refers to the mean of all the data points in each phase. Differences in the means between phases are the first indication of a treatment effect. Next, we will inspect the slope or trend in each phase. An upward trend in symptom severity during the baseline phase vs. a downward trend during the treatment phase would be the strongest indicator of a treatment effect. Finally, we will look at the latency of change. In general, effects occurring closer to the start of the treatment phase would be a strong indicator of a direct treatment effect. Effects occurring more distal to the start of the treatment phase could potentially be due to other factors and would be considered weaker evidence for a treatment effect [40]. In the current study, we expect a delayed treatment effect since the most effective parts of the CBT-I sleep restriction and stimulus control are introduced in the third session. Therefore, the latency of change will be inspected exploratively, starting at session three.

Second, we will use Tau-U to quantitatively assess the differences between the baseline and intervention phase at an individual level. Tau-U is a nonoverlap index (between the phases) that corrects for possible trends in the data [41]. Hereafter, a randomization test will be used to assess the overall effect of the intervention. The randomization test is based on the random assignment of baseline length (i.e., moment of phase change; [42]). This non-parametric test compares the mean difference of all possible baselines with the mean difference between the actual baseline and treatment phase. The place in the rank order of the mean differences determines the p-value of the test. The null hypothesis is that there is no difference between the baseline and treatment phases. The alternative hypothesis is that there is a difference between the two phases. To check for the potential effects of receiving treatment through telehealth versus face-to-face treatment, we will also run the randomization tests for the two treatment options separately.

## Discussion

This study aims to elucidate the effects of an innovative approach to targeting PGD by offering CBT-I for people dealing with both insomnia and PGD. The relationship between prolonged grief symptoms and insomnia symptoms has been well established (see, e.g., [13–16]). To our knowledge, only one small-scale study has examined the effect of CBT-I on prolonged grief symptoms [21]. Although this study showed promising results, the study was underpowered due to significant drop-out. Thus, the results are uncertain and warrant a reexamination. Furthermore, in this earlier study, the authors did not assess whether participants met the diagnostic criteria for PGD and insomnia disorder. The present study, is the first direct investigation into the effect of CBT-I on PGD in people dealing with both PGD and insomnia disorder and uses a design specifically suited for small samples.

Although little experimental evidence exists for the hypothesized effect of CBT-I on PGD, the effects of CBT-I on other affective and stress-related disorders has been well documented (for a review see: [43]). For example, sleep problems have been identified as a risk factor for relapse in depression [44] and targeting sleep problems may prevent the development of major depressive disorder (MDD; [45]). Treating sleep problems co-occurring with depression and PTSD symptomatology has been shown to not only diminish sleep problems but also ameliorate depressive and PTSD symptomatology ([46,47]; for a review, see [43]). Thus, it is not unlikely that similar effects will occur when applying sleep therapy to people with insomnia disorder and PGD.

Furthermore, it is important to understand whether evidence-based CBT-I is effective in the bereaved population. Untreated insomnia puts people at risk for several other affective and stress-related disorders, including depression [44,48], and physical disorders, such as heart diseases (e.g., [49]). Establishing the effects of CBT-I in this population may thus be helpful to improve mental and physical health within the bereaved population, especially considering that the bereaved population experiences more physical problems than their non-bereaved counterparts (e.g., [50]).

It is important to acknowledge that the current study has some potential limitations and bottlenecks. CBT-I is an intensive treatment that can be physically demanding and requires a high level of commitment from the patient [51]. However, it does not include emotionally taxing techniques such as exposure to reminders of the death of the loved one, which is central to CBT for PGD [9]. Thus, it is likely less emotionally overwhelming than CBT for prolonged grief, potentially leading to lower drop-out.

Next, although the study design requires a limited number of participants, the included participants are required to complete weekly questionnaires over the course of six months. This intensive monitoring could potentially result in a higher level of drop-out compared to a study design in which participants are required to complete questionnaires fewer times. We will try to address this problem by replacing dropouts, providing both monetary incentives for each weekly questionnaire as well as by sending reminders, a strategy that has previously been employed successfully, resulting in high completion rates [52].

Additionally, by design, both the participants and the researcher/therapist cannot be blinded, and thus, there is a potential for bias. For example, participants who are familiar with the goal of the study might be inclined to report favorable outcomes. Finally, we will use a convenience sample which might limit the generalizability of the findings.

Notwithstanding these limitations, the study will provide valuable insights into the effect of CBT-I in people with co-occurring insomnia and PGD. This could potentially set the stage for a less emotionally demanding, alternative treatment approach for PGD.

## Supporting information

S1 Checklist Spirit checklist. (DOCX)

S1 File Onderzoeksprotocol versie 3 dd. 03062025. (PDF)
