# Supplementary material for: Treating prolonged grief disorder with CBT for insomnia: A replicated single-case experimental study protocol
Source: PLoS One. 2026 Feb 12;21(2):e0341802. doi: 10.1371/journal.pone.0341802 (PMC12900316; doi:10.1371/journal.pone.0341802)
Supplement: S1 File — (PDF) [file pone.0341802.s002.pdf]

C1 RESEARCH PROTOCOL

Sleep Therapy for People with Prolonged Grief Disorder and Insomnia Disorder

03-06-2025

PROTOCOL TITLE '*Sleep therapy for people with prolonged grief disorder and insomnia disorder*'

**Protocol ID**	**NL86238.042.24**
**Short title**	**Sleep therapy in PGD**
**Version**	**3**
**Date**	**03-06-2025**
**Coordinating investigator/project leader**	***Thomas A de Lang MSc.***<br>***University of Groningen***<br>***Clinical Psychology and Experimental Psychopathology***<br>***Grote Kruisstraat 2/1, 9712 TS Groningen***<br>***email: t.a.de.lang@rug.nl***
**Principal investigator(s) (in Dutch: hoofdonderzoeker/ uitvoerder)**	***Dr. M.C. Eisma***<br>***University of Groningen***<br>***Clinical Psychology and Experimental Psychopathology***<br>***Grote Kruisstraat 2/1, 9712 TS Groningen***<br>***email: m.c.eisma@rug.nl***
**Sponsor (in Dutch: verrichter/opdrachtgever)**	***University of Groningen***<br>***Clinical Psychology and Experimental Psychopathology***<br>***Grote Kruisstraat 2/1, 9712 TS Groningen***
**Subsidising party**	***Stichting tot steun VCVGZ***
**Independent expert (s)**	***Dr. John Krakeel***<br>***GGZ Drenthe***<br>***Dennenweg 9, 9404 LA Assen***<br>***john.krakeel@ggzdrenthe.nl***<br>***BIG- nummer: 39035840901***

TABLE OF CONTENTS

1. INTRODUCTION AND RATIONALE 10
2. OBJECTIVES 11
3. STUDY DESIGN 11
4. STUDY POPULATION 13
   4.1 Population (base) 13
   4.2 Inclusion criteria 13
   4.3 Exclusion criteria 13
   4.4 Sample size calculation 14
5. TREATMENT OF SUBJECTS 15
   5.1 Investigational product/treatment 15
   5.2 Use of co-intervention (if applicable) 15
   5.3 Escape medication (if applicable) 15

Not applicable 15

6. INVESTIGATIONAL PRODUCT 16
   6.1 Name and description of investigational product(s) 16
   6.2 Summary of findings from non-clinical studies 16
   6.3 Summary of findings from clinical studies 16
   6.4 Summary of known and potential risks and benefits 16
   6.5 Description and justification of route of administration and dosage 16
   6.6 Dosages, dosage modifications and method of administration 16
   6.7 Preparation and labelling of Investigational Medicinal Product 16
   6.8 Drug accountability 16
7. NON-INVESTIGATIONAL PRODUCT 17
   7.1 Name and description of non-investigational product(s) 17
   7.2 Summary of findings from non-clinical studies 17
   7.3 Summary of findings from clinical studies 17
   7.4 Summary of known and potential risks and benefits 17
   7.5 Description and justification of route of administration and dosage 17
   7.6 Dosages, dosage modifications and method of administration 17
   7.7 Preparation and labelling of Non Investigational Medicinal Product 17
   7.8 Drug accountability 17
8. METHODS 18
   8.1 Study parameters/endpoints 18
      8.1.1 Main study parameter/endpoint 18
      8.1.2 Secondary study parameters/endpoints (if applicable) 18
      8.1.3 Other study parameters (if applicable) 18
   8.2 Randomisation, blinding and treatment allocation 18
   8.3 Study procedures 18
   8.4 Withdrawal of individual subjects 20
      8.4.1 Specific criteria for withdrawal (if applicable) Not applicable 20
   8.5 Replacement of individual subjects after withdrawal 20

8.6 Follow-up of subjects withdrawn from treatment 20
8.7 Premature termination of the study 20
9. SAFETY REPORTING 21
9.1 Temporary halt for reasons of subject safety 21
9.2 AEs, SAEs and SUSARs 21
9.2.1 Adverse events (AEs) 21
9.2.2 Serious adverse events (SAEs) 21
9.2.3 Suspected unexpected serious adverse reactions (SUSARs) 22
9.2.4 Not applicable. 22
9.3 Annual safety report 22
9.4 Not applicable. 22
9.5 Follow-up of adverse events 22
9.6 Data Safety Monitoring Board (DSMB) / Safety Committee 22
10. STATISTICAL ANALYSIS 23
10.1 Primary study parameter(s) 23
10.2 Secondary study parameter(s) 24
10.3 Other study parameters 24
10.4 Interim analysis (if applicable) 24
Not applicable 24
11. ETHICAL CONSIDERATIONS 25
11.1 Regulation statement 25
11.2 Recruitment and consent 25
11.3 Objection by minors or incapacitated subjects (if applicable) 25
11.4 Benefits and risks assessment, group relatedness 25
11.5 Compensation for injury 26
11.6 Incentives (if applicable) 26
12. ADMINISTRATIVE ASPECTS, MONITORING AND PUBLICATION 27
12.1 Handling and storage of data and documents 27
12.2 Monitoring and Quality Assurance 27
12.3 Amendments 27
12.4 Annual progress report 27
12.5 Temporary halt and (prematurely) end of study report 27
12.6 Public disclosure and publication policy 28
13. STRUCTURED RISK ANALYSIS 29
13.1 Potential issues of concern 29
13.2 Synthesis 29
14. REFERENCES 30

LIST OF ABBREVIATIONS AND RELEVANT DEFINITIONS

AE	Adverse Event
AR	Adverse Reaction
CBT	Cognitive Behavioural Therapy
CBT-I	Cognitive Behavioural Therapy for Insomnia
CV	Curriculum Vitae
DSMB	Data Safety Monitoring Board
GDPR	General Data Protection Regulation; in Dutch: Algemene Verordening Gegevensbescherming (AVG)
IC	Informed Consent
METC	Medical research ethics committee (MREC); in Dutch: medisch-ethische toetsingscommissie (METC)
PGD	Prolonged Grief Disorder
(S)AE	(Serious) Adverse Event
Sponsor	The sponsor is the party that commissions the organisation or performance of the research, for example a pharmaceutical company, academic hospital, scientific organisation or investigator. A party that provides funding for a study but does not commission it is not regarded as the sponsor, but referred to as a subsidising party.
SUSAR	Suspected Unexpected Serious Adverse Reaction
UAVG	Dutch Act on Implementation of the General Data Protection Regulation; in Dutch: Uitvoeringswet AVG
VCVGZ	Vereniging voor Christelijke Verzorging van Geestes- en Zenuwzieken
WMO	Medical Research Involving Human Subjects Act; in Dutch: Wet Medisch-wetenschappelijk Onderzoek met Mensen

SUMMARY

Rationale:

Recent studies provided evidence that insomnia symptoms in bereaved people may constitute an important (causal) risk factor for severe and persistent grief, termed prolonged grief. This strongly suggests that insomnia is a relevant treatment target for prolonged grief disorder. However, high-quality clinical experiments targeting insomnia in people with prolonged grief disorder and comorbid insomnia are lacking. We expect that cognitive behavioral therapy for insomnia (CBT-I) in people with both prolonged grief disorder and insomnia disorder will reduce both symptoms of insomnia and symptoms of prolonged grief.

Objective:

The primary objective is to evaluate whether CBT-I in people with both insomnia and prolonged grief disorder alleviates prolonged grief symptoms. The secondary objective is to evaluate whether CBT-I is an effective treatment of insomnia in people with comorbid prolonged grief disorder.

Study design:

We plan to conduct a replicated two-phase single-case experimental design study, testing the efficacy of CBT-I in participants with prolonged grief disorder and comorbid insomnia. We choose this design as it offers the possibility to study causal mechanisms with a limited sample size.

Participants can enroll for the study through our website (www.onderzoekrouw.nl). They are invited to complete a screening questionnaire for prolonged grief disorder and insomnia disorder. Participants who score above the established cut-off scores on the Insomnia Severity Inventory (ISI) and the Traumatic Grief Inventory Self-Report Plus (TGI-SR+) will be invited for a diagnostic interview. Following the diagnostic interview, the baseline phase (waiting period) will start. Participants will be randomly assigned to different lengths of waiting periods based on a computer-generated list. The baseline phase can range between five and 14 weeks. During this phase, participants will be asked weekly, to complete a short online questionnaire on insomnia (ISI) and PGD symptoms (TGI-SR+) experienced during the prior week. The weekly assessment of insomnia and PGD symptoms will continue during the treatment phase. Insomnia and prolonged grief symptoms will also be reassessed at a three-month post-treatment follow-up.

Study population:

Twenty adult participants (18 years or older) meeting the DSM-5-TR criteria for both prolonged grief disorder and insomnia disorder.

Intervention (if applicable):

We will provide all participants with CBT-I, the first-choice treatment for insomnia (Riemann et al., 2023). CBT-I consists of 6-7 weekly individual, face-to-face sessions of 1-2 hours

according to the Dutch CBT-I protocol (Verbeek & van de Laar, 2023). Depending on the location of the participant, these sessions can be held either at the Heymans Institute or online using a video conference program. At four weeks post-treatment, a booster session will be planned to evaluate sleep health and, if necessary, repeat certain treatment components. Prior to CBT-I, participants will be randomly assigned to waiting periods between five and 14 weeks (the baseline phase).

Main study parameters/endpoints:

The main parameters of the study are: changes in prolonged grief symptom severity between baseline and treatment phase, as measured by the TGI-SR+ and changes in insomnia symptom severity between baseline and treatment phase, as measured by the ISI.

Nature and extent of the burden and risks associated with participation, benefit and group relatedness:

The current study will be of minimal burden and negligible risk to participants. Exclusion criteria are used to minimize risks to participants. In addition to the clinical interview at the start of the study, participants will be asked to fill out 26 weekly questionnaires consisting of 19 multiple-choice questions (< 5 minutes to complete) for which they will receive 5 euros per assessment. These questionnaires will also be used as an evaluation of the treatment. Finally, participants will be asked to complete a follow-up questionnaire, three months after the last treatment session. This questionnaire will take no more than 5 minutes to complete and results in an additional payment of 50 euros.

There are minimal risks/burden associated with CBT-I. CBT-I is the gold standard treatment for insomnia worldwide, has been investigated in numerous studies, and is recommended in clinical practice (see: Riemann et al., 2023). Furthermore, CBT-I has been shown to be effective in people with comorbid psychiatric disorders (see also: Mijnster et al., 2022)

An important component of CBT-I is stimulus control therapy. During stimulus control therapy participants are instructed to leave the bedroom when noticing they are not falling asleep and to return only when feeling sleepy. Furthermore, participants are instructed to refrain from any activity other than sleep and sex in the bedroom. For elderly mobility-impaired participants, getting out of bed during nighttime might increase the risk of falling. These participants are therefore not instructed to get out of bed. These participants will be instructed to remain in bed, and to do something none sleep related and relaxing such as listing to a podcast, or reading a magazine, in low-light conditions.

Another important component of CBT-I is sleep restriction therapy, in which patients are instructed to restrict their bedtimes to their average sleep time. Reducing sleep times could lead to certain risks. Specifically, this intervention is associated with mild short-term side effects such as performance impairment and reduced reaction times (Kyle et al., 2014).

These side effects are similar to what is normally observed in insomnia patients. Therefore, for reasons of safety, participants are instructed to spend a minimum of 5 hours in bed each night and to refrain from driving a car or operating heavy machinery if they feel excessively sleepy during the early stages of sleep restriction therapy. Importantly, sleep restriction is a potent intervention technique that may lead to some side effects at first, but also leads to an amelioration of such experiences over time.

Additionally, minimal risks/burden are associated with the repeated measurement of symptoms of prolonged grief disorder and insomnia. This repeated measurement might elicit some negative feelings in participants. However, it is our experience from prior survey research among hundreds of bereaved adults, that they generally find it comforting to know that their grief can contribute to something positive, such as scientific knowledge. Nevertheless, to further reduce the risk of eliciting negative feelings, we will only use validated questionnaires applied successfully before in the bereaved population.

Finally, participants who still experience severe and disabling grief reactions (TGI-SR + score $\geq$ 33, on the DSM5-TR items) at the 3-month follow-up will be offered the possibility of receiving CBT for prolonged grief disorder from a psychologist within the research team. CBT for prolonged grief disorder is the golden standard treatment for prolonged grief disorder and can be effectively delivered in person and online (e.g., Komischke-Konnerup et al., 2024; Wagner et al., 2020).

1. INTRODUCTION AND RATIONALE

A substantial minority of bereaved adults experiences persistent, severe and disabling grief, also termed prolonged grief (10%; Lundorff et al., 2017). Prolonged Grief Disorder (PGD), characterized by such grief responses, is newly included in the Diagnostical and Statistical Manual of Mental Disorders (5th edition, text revision, American Psychiatric Association, 2022). Core symptoms include persistent and severe yearning and/or cognitive preoccupation with the deceased, combined with symptoms indicative of emotional pain, experienced more than 12 months after loss.

Randomized controlled trials of treatments for PGD, such as cognitive behaviour therapy, result in clinically relevant reductions of PGD symptoms in at most half of clients and effect sizes are on average moderate (Doering & Eisma, 2016; Johannsen et al., 2019). Therefore, there is a clear need to improve existing treatment options for PGD. We posit that targeting insomnia (i.e., long-lasting (≥ 3 months) and frequent (≥ 3 nights a week) problems with falling and/or staying asleep, causing daytime distress/impairments) (American Psychiatric Association, 2022), could be a viable new treatment option for PGD, for multiple reasons.

First, sleep plays a critical role in emotion regulation. During sleep, particularly rapid-eye movement sleep (REM-sleep), emotional events are reactivated, reprocessed and reconsolidated. Insomnia is typically associated with fragmented (‘restless’) REM-sleep, which may disrupt the overnight adaptation to experienced negative emotions and distress (e.g., Riemann et al., 2012; Vandekerckhove & Wang, 2018).

Second, sleep problems are common among people with mental disorders in general (Freeman et al., 2020) and PGD in particular (Lancel et al., 2020). Poor sleep negatively impacts stress-related and affective disorders. Insomnia increases the risk for the future development of a major depressive disorder (MDD) and posttraumatic stress disorder (PTSD) (Hertenstein et al., 2023). Furthermore, evidence-based insomnia treatments, such as cognitive behavioural treatment of insomnia (CBT-I), not only improve sleep, but also prevent the development of MDD (Irwin et al., 2022) and ameliorate depressive and PTSD symptomatology (Gee et al., 2019; Ho et al., 2016). This suggests that sleep problems constitute a transdiagnostic risk factor and that targeting these could similarly be effective for people with PGD and comorbid insomnia.

Third, a recent systematic review showed that sleep problems are often comorbid with PGD and do not fully remit after psychotherapy for PGD (Lancel et al., 2020). Building on these findings, we established that among recently bereaved adults, three trajectories of insomnia symptoms emerge: chronic (persistent clinical levels of insomnia), recovering (sub-clinical levels of insomnia that reduce over time), and resilient (non-clinical levels of insomnia that reduce over time). Trajectories of insomnia symptoms strongly related to trajectories of prolonged grief symptoms and probable PGD was most prevalent in people with a chronic insomnia trajectory (de Lang et al., 2024). Moreover, we demonstrated that changes in insomnia symptoms are predictive of changes in prolonged grief symptoms, but not vice versa (de Lang et al., 2023). In summary, findings clearly point to insomnia as a promising treatment target for people with PGD.

2. OBJECTIVES

Primary Objective: The primary objective is to establish whether CBT-I reduces PGD symptoms in people with PGD and insomnia
Secondary Objective(s): The secondary objective is to establish whether CBT-I reduces insomnia symptoms in bereaved people with PGD and insomnia.

3. STUDY DESIGN

We will use a replicated two-phase single-case experimental design (SCED) study to determine the efficacy of CBT-I, the first-choice insomnia treatment (Qaseem et al., 2016; Riemann et al., 2023), on PGD (and insomnia) symptoms in persons with PGD and insomnia disorder. We choose replicated SCED, because it offers the possibility to study causal mechanisms with a limited sample size, thereby increasing the feasibility of the study.

A SCED requires continuous repeated measurements both in the baseline and the treatment phase. It also requires randomization of the length of the baseline phase. These different baseline lengths are important because they help map the variability of prolonged grief and insomnia symptoms over time, ensuring that observed changes during the intervention phase are not due to pre-existing trends or fluctuations. Having different starting points provides a robust comparison, allowing us to distinguish between natural fluctuations and the effects of the intervention. Assigning different baseline lengths protects against threats to the internal validity of our study, such as statistical regression to the mean and natural changes that individuals may undergo simply due to repeated measurement or the natural recovery from grief symptoms (Morley, 2018). Unlike traditional group designs such as an RCT, replicated SCED relies on within-subject comparisons; each participant serves as their own control. Replication across multiple individuals strengthens the validity of findings, reducing the likelihood that results are due to chance or individual variability. Additionally, it offers the opportunity to evaluate the effects of an intervention on symptom patterns in individual patients. The replicated SCED is one of the strongest SCED designs as it allows for the control of maturation effects (Maric & van der Werff, 2020).

Following recruitment, participants will be randomly assigned based on a computer-generated list to different lengths of baselines (or wait time), ranging between five and 14 weeks (see Table 1 for an overview). During this phase, participants will be asked to complete weekly short online questionnaires assessing insomnia symptoms (Insomnia Severity Index, ISI: (Morin, 1993; Dutch version: Bastien et al., 2001)) and PGD symptoms (TGI-SR+: Lenferink et al., 2022) experienced during the past week. The weekly assessment of insomnia and PGD symptoms will continue during the treatment phase. The treatment phase consists of 6-7 weekly therapy sessions and at four-week post-treatment a booster session, and continued weekly monitoring of insomnia and PGD symptoms. The continued monitoring allows for the capturing of delayed treatment effects. In order to (visually) inspect long term effects, insomnia and PGD symptoms will be assessed again three-months post-treatment. A weblink to online questionnaires will be sent via e-mail to participants, CBT-I will be delivered by a psychologist in training to be become a healthcare psychologist, supervised by a registered healthcare psychologist, specialised in insomnia. CBT-I consists

of 6-7 weekly individual, face-to-face sessions of 1-2 hours according to the Dutch CBT-I protocol (Verbeek & van de Laar, 2023). Depending on the location of the participant, these sessions can be held either at the Heymans Institute or online using a video conference program. At four weeks post-treatment, a booster session will be planned to evaluate sleep health and, if necessary, repeat certain treatment components.

CBT-I components are:
Psycho-education: providing information about the function and regulation of normal sleep and the consequences of disturbed sleep.
Sleep hygiene: giving information on healthy lifestyle that can improve sleep and on behaviors that disrupt sleep.
Stimulus control: strengthening the association between bed (and bedroom) and sleep by only going to bed when sleepy and leaving the bed when awake for longer than 30 min and getting up at a fixed time.
Sleep restriction therapy: restricting bedtime to the average subjective total sleep duration during the past week (≥ 5 hours) and thereby elevating sleep pressure, which will result in a higher relative percentage of time slept while in bed (sleep efficiency). Based on reported sleep efficiency during the past week and the participant's age bedtimes will be extended or reduced.
Relaxation therapy: employing various relaxation techniques to teach a person to unwind.
Cognitive therapy: identifying and challenging misconceptions and worries about sleep that keep a person awake.

To ensure retention of the participants throughout the study, participants will be paid €5,- upon completion of each set of questionnaires (26 in total), the follow-up questionnaire will result in an additional payment of €50,-. Travel expenses for visits to the Heymans Institute will be reimbursed.

Table 1. *Overview of three (of ten) possible baseline and treatment phase options.*

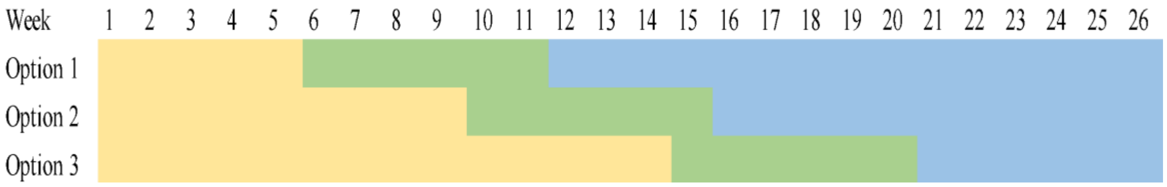

Note: yellow = baseline phase, green= treatment phase (therapy sessions), blue = continued monitoring). A follow-up measurement will be administered at 3 months post-treatment (not included in the table).

4. STUDY POPULATION

4.1 Population (base)

The population consists of Dutch adults who are bereaved of a loved one. Bereavement is a common life experience. Approximately 170.000 people pass away each year in the Netherlands (Centraal Bureau voor Statistiek, 2024). Conservatively estimated, each death leaves around four people bereaved of a loved one, corresponding with around 680.000 bereaved each year. In approximately 10% of the bereaved due to natural causes, the loss results in severe, persistent, and disabling grief reactions, also termed prolonged grief. The incidence rate of Dutch adults with prolonged grief disorder, a disorder characterized by such severe, persistent and disabling grief reaction (American Psychiatric Association, 2022) is therefore around 70.000 to 125.000 per year in the Netherlands.

One of the most commonly reported problems following bereavement are sleep disturbances (Monk et al., 2008). The severity of sleep problems and grief reactions are positively correlated (e.g., Lancel et al., 2020). The estimated 5-year incidence rate of insomnia among initially good sleepers is 11 and 17% (Morin et al., 2020), the rate among the bereaved with prolonged grief disorder is likely to be higher (e.g., Boelen & Lancee, 2013; Brown et al., 1996; Monk et al., 2008; see: Lancel et al., 2020). Based on prior studies, we estimate that about half of the bereaved who can be diagnosed with prolonged grief disorder will also fulfil the diagnostic criteria of insomnia disorder. Thus, around 35.000 to 67.000 people per year would qualify for our study.

In a longitudinal study approved by the Ethical Committee Psychology (ECP) of the University of Groningen, we are currently measuring individuals' insomnia and prolonged grief symptoms in over 200 bereaved adults. Within this longitudinal study, participants will be asked to indicate if they are interested in receiving information about the current intervention study (without obligations). Considering that we only require twenty participants to achieve sufficient power for the proposed intervention study, we are confident that we will meet our sample size requirements.

4.2 Inclusion criteria

To be eligible to participate in this study, a subject must complete the screening questionnaire and meet all of the following criteria: - being eighteen years or older and meeting the diagnostic criteria for prolonged grief disorder and insomnia disorder (as specified in the DSM-5-TR).

4.3 Exclusion criteria

Recently started (<3 months) or changed use of sleep medication or off-label sedating medication for insomnia complains, being diagnosed with, but not yet treated for, a different sleep disorder (particularly obstructive sleep apnea syndrome, restless legs syndrome), doing shiftwork, having traveled across time zones during the last week (>4hours time difference), being diagnosed with a schizophrenia spectrum disorder, a substance use disorder, being suicidal, or mental retardation, or being unable to adequately read or speak Dutch will lead to exclusion from the study, to ensure unbiased evaluation of treatment effects (cf. van der Zweerde et al., 2020).

4.4 Sample size calculation

Based on a prior small pilot study on CBT-I effects on prolonged grief symptoms (Sveen et al., 2021) and studies on CBT-I effects on depression symptoms (e.g., Christensen et al., 2016; van der Zweerde et al., 2020), we estimate a large effect size (*Cohen's d* = 1.0) on prolonged grief and insomnia symptoms. An a priori power analysis was conducted using the single case designs shiny web-app for power calculations (Bouwmeester & Jongerling, 2020). Based on power of .80, a total of 26 measurements per participant, of which minimally five during baseline phase, followed by minimally 12 measurements during the treatment (and continued monitoring) phase and an alpha of .05, sample size of 19 is required (see Table 1 for an overview). A total of 20 patients will be enrolled to ensure sufficient power. CBT-I provided generally elicits a low dropout rate (e.g., Lancee et al., 2016), in case a participant drops out, they will be replaced by another participant.

5. TREATMENT OF SUBJECTS

5.1 Investigational product/treatment

CBT-I will be delivered by a psychologist in training to become a healthcare psychologist, supervised by a registered healthcare psychologist. CBT-I consists of 6-7 weekly individual, face-to-face sessions of 1-2 hours according to the Dutch CBT-I protocol (Verbeek & van de Laar, 2023). Depending on the location of the participant, these sessions can be held either at the Heymans Institute or online using a video conference program. At four weeks post-treatment, a booster session will be planned to evaluate sleep health and, if necessary, repeat certain treatment components.

Components of CBT-I are:
Psycho-education: providing information about the function and regulation of normal sleep and the consequences of disturbed sleep.
Sleep hygiene: giving information on healthy lifestyle that can improve sleep and on behaviors that disrupt sleep.
Stimulus control: strengthening the associations between bed (and bedroom) and sleep by only going to bed when sleepy and leaving the bed when awake for longer than 30 min, and getting up at a fixed time and refraining from any activity other than sleep and sex in the bedroom. For mobility-impaired participants, getting out of bed during nighttime might increase the risk of falling. These participants are therefore not instructed to get out of bed. These participants will be instructed to remain in bed, and do something none sleep related and relaxing such as listing to a podcast, or reading a magazine, in low light conditions.
Sleep restriction therapy: restricting bedtime to the average subjective total sleep duration during the past week (≥ 5 hours) and thereby elevating sleep pressure, which will result in a higher relative percentage of time slept while in bed (sleep efficiency). Based on reported sleep efficiency during the past week and participant age, bedtimes will be extended or reduced.
Relaxation therapy: employing various relaxation techniques to teach a person to unwind.
Cognitive therapy: identifying and challenging misconceptions and worries about sleep that keep a person awake.

5.2 Use of co-intervention (if applicable)

Participants will be instructed not to seek additional psychological counselling for prolonged grief disorder during the study. They will be allowed to seek psychological care for other psychological disorders. Participants will be asked at the start of the study and after completion if they have received any additional care.

5.3 Escape medication (if applicable)

Not applicable

6. INVESTIGATIONAL PRODUCT

6.1 Name and description of investigational product(s)

6.2 Summary of findings from non-clinical studies

6.3 Summary of findings from clinical studies

6.4 Summary of known and potential risks and benefits

6.5 Description and justification of route of administration and dosage

6.6 Dosages, dosage modifications and method of administration

6.7 Preparation and labelling of Investigational Medicinal Product

6.8 Drug accountability

7. NON-INVESTIGATIONAL PRODUCT

Not applicable.

7.1 Name and description of non-investigational product(s)

7.2 Summary of findings from non-clinical studies

7.3 Summary of findings from clinical studies

7.4 Summary of known and potential risks and benefits

7.5 Description and justification of route of administration and dosage

7.6 Dosages, dosage modifications and method of administration

7.7 Preparation and labelling of Non Investigational Medicinal Product

7.8 Drug accountability

8. METHODS

8.1 Study parameters/endpoints

Clinical interviews and self-report questionnaires assessing DSM-5-TR insomnia and prolonged grief symptoms will be administered at the start of the study. Insomnia and prolonged grief symptoms will be measured weekly for a period of 26 weeks using self-report questionnaires.

8.1.1 Main study parameter/endpoint

The primary aim is to assess the effect of CBT-I on prolonged grief symptoms. To assess this effect, prolonged grief symptoms will be measured weekly for a period of 26 weeks starting parallel to the waiting period and continuing through the treatment phase. The mean difference in symptom levels between the baseline phase (waiting period) and treatment phase will serve as the primary outcome.

8.1.2 Secondary study parameters/endpoints (if applicable)

The secondary aim is to assess the effect of CBT-I on insomnia symptoms in people with prolonged grief disorder. The analysis of the insomnia symptoms will be identical to the analysis of prolonged grief symptoms.

8.1.3 Other study parameters (if applicable)

Besides primary and secondary outcome measures, inclusion and exclusion criteria will be assessed next to sociodemographic and loss-related characteristics (age, gender, education level, kinship to the deceased, gender of the deceased, cause of death, expectedness of the loss).

8.2 Randomisation, blinding and treatment allocation

Randomization will be used to determine the length of the waiting period (the baseline). We will use randomizer.org to generate a random list of 20 numbers ranging between five and 14, representing the possible number of weeks of the waiting period. Directly after completing the clinical interview, the participant will be allocated to the first available number on this computer-generated list. In case drop-out occurs a new number between five and 14 will be drawn and added to the end of the list.

8.3 Study procedures

Recruitment of bereaved adults (≥ 18 years) who lost a first-degree relative or partner will take place via localized Google AdWords advertisements and websites for organizations for bereaved persons, linking to a research website (www.onderzoekrouw.nl). Additionally, people who have participated in earlier studies and indicated an interest in future studies will be asked to visit the website. On the website, interested bereaved adults can access information about the study (see E1) and can provide informed consent on a secure online survey program, Qualtrics. After providing online informed consent (see E2), they will be requested to complete a screener for sociodemographic and loss-related characteristics, sleep disorders (ISI; Bastien et al., 2001; Holland Sleep Disorders Questionnaire; HSDQ; Kerkhof et al., 2013) and PGD (Traumatic Grief Inventory Self-Report Plus; TGI-SR+:

Lenferink et al., 2022).

People who meet the established cut-off values for insomnia disorder (ISI score >14) and prolonged grief disorder (TGI-SR+ score ≥ 33, on the DSM5-TR items) based on screener results will be invited to meet online (via a secure online meeting program) or to visit the Heymans Institute of the University of Groningen for a clinical interview to assess PGD (Traumatic Grief Inventory-Clinician Administered, TGI-CA; Lenferink et al., 2023) and insomnia disorder (Structured Clinical Interview for DSM-5 Sleep Disorders, SCIDS; Taylor et al., 2018). The diagnostic criteria for PGD are intense yearning/longing for the deceased and/or preoccupation with thoughts or memories of the deceased, combined with three or more emotional, cognitive, and behavioral symptoms (DSM5-TR; American Psychiatric Association, 2022). The diagnostic criteria for insomnia disorder are experiencing problems falling and/or staying asleep at least 3 nights per week and for at least 3 months resulting in daytime dysfunction/distress (DSM5-TR; American Psychiatric Association, 2022).

Prior to the clinical interviews, participants will be provided with the study information letter once more (see E1) and an informed consent form (see E2). The clinical interviews will be conducted by a psychologist in training to become a registered healthcare psychologist under the supervision of a registered healthcare psychologist. When participants meet all inclusion criteria and none of the exclusion criteria, they can immediately start the waiting period for CBT-I.

Once the waiting period has started, participants will receive weekly questionnaires assessing prolonged grief (TGI-SR+) and insomnia symptoms (ISI). These weekly questionnaires will continue for 26 weeks covering both the baseline and intervention phase of the study.

Following the waiting period, participants will receive CBT-I following the standard Dutch protocol (Verbeek & van de Laar, 2023). CBT-I will be delivered by a psychologist in training to become a healthcare psychologist, supervised by a registered healthcare psychologist. CBT-I consists of 6-7 weekly individual, face-to-face sessions of 1-2 hours according to the Dutch CBT-I protocol (Verbeek & van de Laar, 2023). Depending on the location of the participant, these sessions can be held at the Heymans Institute or online using a video conference program. At four weeks post-treatment, a booster session will be planned to evaluate sleep health and, if necessary, repeat certain treatment components.

Measures used during the study

- The Holland Sleep Disorder Questionnaire (HSDQ; Kerkhof et al., 2013) is a validated, Dutch, self-assessment questionnaire to screen for sleep disorders, such as insomnia disorder, sleep-related breathing disorders, and sleep-related movement disorders.
- The Traumatic Grief Inventory Plus (TGI-SR+; Lenferink et al., 2022) is a validated questionnaire that assesses all current symptoms of disturbed grief, including PGD as per ICD-11 and DSM5-TR. We will use the 12 items reflecting symptoms of DSM-5-TR PGD. We will use the original version, assessing symptoms over the past month, as well as an adapted version assessing symptoms over the past week.
- The Traumatic Grief Inventory Clinician Administered (TGI-CA; Lenferink et al., 2023) is an interview to assess all current criteria for disturbed grief, including PGD per ICD-11 and DSM-5-TR. We will use the 12 items reflecting symptoms of DSM-5-TR PGD.
- The Structured Clinical Interview for DSM5 Sleep Disorders Revised (SCISD-R; Taylor et al., 2018) is a well-validated clinical interview to assess DSM-5-TR sleep disorders, including insomnia disorder.

- The Insomnia Severity Index (ISI; Bastien et al., 2001) is a widely used and validated questionnaire that assesses insomnia severity. Items cover both day- and nighttime symptoms as well as worries about sleep. The ISI will be slightly adjusted to assess symptoms in the last week.

8.4 Withdrawal of individual subjects

Subjects can leave the study at any time without providing any reason if they wish to do so without any consequences.

8.4.1 Specific criteria for withdrawal (if applicable)

Not applicable

8.5 Replacement of individual subjects after withdrawal

If for any reason participants decide to withdraw from participation in the study before completing or during CBT-I, new participants will be included to ensure sufficient power for conducting the planned analyses.

8.6 Follow-up of subjects withdrawn from treatment

Participants who withdraw from the study will be invited to complete all assessments.

8.7 Premature termination of the study

If unexpected side effects or events occur that the researchers have not foreseen and are possibly associated with the proposed intervention, the study will be ended prematurely.

9. SAFETY REPORTING

9.1 Temporary halt for reasons of subject safety

In accordance to section 10, subsection 4, of the WMO, the sponsor will suspend the study if there is sufficient ground that continuation of the study will jeopardise subject health or safety. The sponsor will notify the accredited METC without undue delay of a temporary halt including the reason for such an action. The study will be suspended pending a further positive decision by the accredited METC. The investigator will take care that all subjects are kept informed.

9.2 AEs, SAEs and SUSARs

9.2.1 Adverse events (AEs)

Adverse events are defined as any undesirable experience occurring to a subject during the study, whether or not considered related to the experimental intervention. All adverse events reported spontaneously by the subject or observed by the investigator or his staff will be recorded.

9.2.2 Serious adverse events (SAEs)

A serious adverse event is any untoward medical occurrence or effect that
results in death; is life threatening (at the time of the event); requires hospitalisation or prolongation of existing inpatients' hospitalisation; results in persistent or significant disability or incapacity; is a congenital anomaly or birth defect; or any other important medical event that did not result in any of the outcomes listed above due to medical or surgical intervention but could have been based upon appropriate judgement by the investigator.
An elective hospital admission will not be considered as a serious adverse event. The investigator will report all SAEs to the sponsor without undue delay after obtaining knowledge of the events.

The sponsor will report the SAEs through the web portal *ToetsingOnline* to the accredited METC that approved the protocol, within 7 days of first knowledge for SAEs that result in death or are life threatening followed by a period of maximum of 8 days to complete the initial preliminary report. All other SAEs will be reported within a period of maximum 15 days after the sponsor has first knowledge of the serious adverse events.

9.2.3 Suspected unexpected serious adverse reactions (SUSARs)

9.2.4 Not applicable.

9.3 Annual safety report

9.4 Not applicable.

9.5 Follow-up of adverse events

All AEs will be followed until they have abated, or until a stable situation has been reached. Depending on the event, follow-up may require additional tests or medical procedures as indicated, and/or referral to the general physician or a medical specialist.
SAEs need to be reported till end of study within the Netherlands, as defined in the protocol

9.6 Data Safety Monitoring Board (DSMB) / Safety Committee

Based on the nature of this study, a Data Safety Monitoring Board (DSMB) is considered unnecessary. According to the NFU Risk Classification and Monitoring, the risk of this study is negligible. Therefore, there is no need to perform interim analyses concerning safety and effectiveness of the treatment during the course of this trial. An independent monitor will be involved in this study.

10. STATISTICAL ANALYSIS

Data analyses will be based on intention-to-treat analyses and will be performed using R software. Descriptive statistics will be described in frequency tables or presented as means and standard deviations.

10.1 Primary study parameter(s)

The primary outcome of the study is prolonged grief symptoms as measured by the TGI-SR+. To test the hypothesis that prolonged grief symptoms will decline during the treatment of comorbid insomnia we will use two analytic strategies. First, we will use visual inspection. The data of each participant will be plotted separately. We will then inspect changes in the mean level between the baseline phase and the treatment phase. The mean level refers to the mean of all the data points in each phase. Differences in the mean are the first indication of a treatment effect. Next, we will inspect the slope or trend in each phase. An upward trend in symptom severity during the baseline phase vs. a downward trend during the treatment phase would be the strongest indicator of a treatment effect. Finally, we will look at the latency of change. In general, effects occurring closer to the start of the treatment phase would be a strong indicator of a direct treatment effect. Effects occurring more distal to the start of the treatment phase could potentially be due to other factors and would be considered weaker evidence for a treatment effect (Kazdin, 2019). In the current study, we expect a delayed treatment effect since the most effective parts of the CBT-I sleep restriction and stimulus control are introduced in the second session. Therefore, the latency of change will be looked at exploratively.

Second, we will use Tau-U to quantitatively assess the differences between the baseline and intervention phase at an individual level. Tau-U is a non-overlap index (between the phases) that corrects for possible trends in the data (Parker et al., 2011). Hereafter, a randomization test will be used to assess the overall effect of the intervention. The randomization test is based on the random assignment of baseline length (i.e., moment of phase change; (Michiels & Onghena, 2019). This non-parametric test compares the mean difference of all possible baselines (10 per person in the current study) with the mean difference between the actual baseline and treatment phase. The place in the rank order of the mean differences determines the p-value of the test. The null hypothesis is that there is no difference between the baseline and treatment phases. The alternative hypothesis is that there is a difference between the two phases. To check for the potential effects of receiving treatment through telehealth versus face-to-face treatment, we will also run the randomization tests for the two treatment options separately.

10.2 Secondary study parameter(s)

The secondary study outcome are insomnia symptoms as measured by the ISI. The analysis of the insomnia symptoms will be identical to the analysis of prolonged grief symptoms described above.

10.3 Other study parameters

Sociodemographic and loss-related characteristics (age, gender, education level, kinship to the deceased, gender of the deceased, cause of death, expectedness of the loss) clinical characteristics at baseline will be used to describe the sample.

10.4 Interim analysis (if applicable)

Not applicable

11. ETHICAL CONSIDERATIONS

11.1 Regulation statement

This study will be conducted according to the principles of the Declaration of Helsinki (2013) and in accordance with the Medical Research Involving Human Subjects Act (WMO). Moreover, the General Data Protection Regulation (GDPR in Dutch: Uitvoeringswet Algemene Verordering Gegevensbescherming)

11.2 Recruitment and consent

Recruitment of bereaved adults (≥ 18 years) who lost a first-degree relative or partner will take place via localized Google AdWords advertisements and websites for organizations for bereaved persons, linking to a research website (www.onderzoekrouw.nl). Additionally, people who have participated in an earlier longitudinal survey and indicated an interest in future studies will be asked to visit the website. On the website, interested bereaved adults can access information about the study (see E1) and can provide informed consent on a secure online survey program, Qualtrics. After providing online informed consent (see E2), they can complete a screener for sociodemographic and loss-related characteristics, sleep disorders (HSDQ; Kerkhof et al., 2013) and PGD (TGI-SR+; Lenferink et al., 2022).

People who meet the established cut-off values for insomnia disorder and PGD based on screener results, will be invited to meet online (via a secure online meeting program) or to visit the Heymans Institute of the University of Groningen for a clinical interview to assess PGD (Traumatic Grief Inventory-Clinician Administered, TGI-CA; Lenferink et al., 2023) and insomnia disorder (Structured Clinical Interview for DSM-5 Sleep Disorders, SCISD; Taylor et al., 2018).

Prior to the clinical interviews, participants will be provided with the study information letter once more (see E1) and an informed consent form (E2) to provide written informed consent. When participants meet all inclusion criteria and none of the exclusion criteria, they can start the waiting period. Before the start of the clinical interview, participants will have the opportunity to ask any questions or concerns they may have to the researcher that is present. In addition, the information letter (E1) notes that participants may ask any questions they may have by email.

11.3 Objection by minors or incapacitated subjects (if applicable)

Not applicable.

11.4 Benefits and risks assessment, group relatedness

A benefit of the current study is that it investigates a promising new approach to the treatment of prolonged grief disorder. Current evidence-based treatments for prolonged grief disorder have shown limited success rates, with no more than 50% of clients reporting clinically relevant improvement (for reviews: Doering & Eisma, 2016; Johannsen et al., 2019). Recent studies have suggested that insomnia symptoms might be causally implicated in the persistence of severe, disabling grief reactions (e.g., de Lang et al., 2023, 2024; Lancel et al., 2020). Insomnia therapy has shown promising effects in other stress and mood-related disorders such as depression (Gee et al., 2019) and post-traumatic stress disorder (Ho et al.,

2016) and a small pilot study has shown promising effects of insomnia therapy on prolonged grief symptoms in bereaved parents (Sveen et al., 2021). Therefore, it is likely that targeting insomnia symptoms in people with both prolonged grief disorder and insomnia disorder will reduce both insomnia and prolonged grief symptoms.

A benefit for participants in the study is that we will use CBT-I, the gold standard treatment for insomnia worldwide, which has been investigated in numerous studies, and is recommended in clinical practice (see: Riemann et al., 2023). While CBT-I has minimal risks, some components carry specific concerns. Stimulus control therapy, which involves leaving the bedroom if unable to sleep and using the bed solely for sleep and sex, may increase the risk of falls for elderly or mobility-impaired participants. To prevent this, such participants will not be instructed to leave the bed. Sleep restriction therapy, another CBT-I component, requires limiting time in bed to the average duration of sleep. Although effective, this approach can cause short-term side effects like reduced reaction times and performance impairments, common among those with insomnia. To enhance safety, participants will spend at least five hours in bed each night and will be advised to avoid driving or operating heavy machinery if they feel excessively sleepy during the therapy's early stages. Over time, these side effects typically diminish as the therapy takes effect.

Additionally, the study involves repeated measurement of prolonged grief and insomnia symptoms, which could evoke negative emotions. However, prior research indicates that bereaved participants often find value in contributing to scientific knowledge. To minimize distress, only validated, concise questionnaires with completion times under five minutes will be used. Overall, the study offers the potential for significant improvements in both insomnia and prolonged grief symptoms, with minimal risks.

11.5 Compensation for injury

The researchers asked for dispensation for compulsory insurance in medical research in human subjects, because they do not expect additional risks for the participants (article 7 paragraph 5 WMO). We refer to our discussion in the previous section on benefits and risks. However, the liability of the researcher or provider is assured. The sponsor/investigator has a liability insurance which is in accordance with article 7 of the WMO.

11.6 Incentives (if applicable)

In addition to receiving evidence-based treatment for insomnia disorder, participants will receive 5 euros for each set of completed questionnaires (26 in total) with an extra 50 euro bonus for completion of the follow-up questionnaires.

12. ADMINISTRATIVE ASPECTS, MONITORING AND PUBLICATION

12.1 Handling and storage of data and documents

The researchers will manage data and documents confidentially. Paper documents resulting from this study, such as consent forms, are stored separately in a secured location at the RUG. Contact data will be stored in a digital file for the purpose of administering the assessments at the planned moments. In a key file, each participant is assigned a personal code, which links research data to individual participants. These codes are not based on the initials, birth-date or other participant related characteristics. The code list will start at 101 in ascending order. The coordinating investigator and principal investigator the only persons with access to the key file. In addition, the code list will be stored on Qualtrics our secure online questionnaire partner. This is needed in order to send the questionnaires with participant number to the correct participant. The research data will be processed and analysed in separate files that contain no direct links to the participants. Digital and paper data necessary to reproduce analysis and quality checks will be kept for 15 years at RUG conform local and official regulations (GDPR) as stated in the RUG data storage protocol

12.2 Monitoring and Quality Assurance

The coordinating investigator, principal investigator, and research assistants are the only persons with access to the key file. The research data will be processed and analysed in separate files that contain no direct links to the participants. Digital and paper data necessary to reproduce analysis and quality checks will be kept for 15 years at RUG conform local and official regulations (GDPR) as stated in the RUG data storage protocol

12.3 Amendments

Amendments are changes made to the research after a favourable opinion by the accredited METC has been given. All amendments will be notified to the METC that gave a favourable opinion.

12.4 Annual progress report

The sponsor/investigator will submit a summary of the progress of the trial to the accredited METC once a year. Information will be provided on the date of inclusion of the first subject, numbers of subjects included and numbers of subjects that have completed the trial, serious adverse events/ serious adverse reactions, other problems, and amendments.

12.5 Temporary halt and (prematurely) end of study report

The investigator/sponsor will notify the accredited METC of the end of the study within a period of 8 weeks. The end of the study is defined as the last patient’s follow-up questionnaire is completed or was scheduled but not completed within a month.
The sponsor will notify the METC immediately of a temporary halt of the study, including the reason of such an action.

In case the study is ended prematurely, the sponsor will notify the accredited METC within 15 days, including the reasons for the premature termination.
Within one year after the end of the study, the investigator/sponsor will submit a final study report with the results of the study, including any publications/abstracts of the study, to the accredited METC.

12.6 Public disclosure and publication policy

The proposed study will result in two English language open access peer-reviewed scientific papers (one study protocol paper or trial registration, one primary outcome paper). We plan to publish a Dutch translation of our primary outcome paper in Gedragstherapie, a journal for Dutch cognitive-behaviour therapists. Moreover, we will present the results of the study at (minimally) two (inter)national conferences, such as the European Grief Conference and the VCGT Najaarscongres.

13. STRUCTURED RISK ANALYSIS

Not applicable

13.1 Potential issues of concern

13.2 Synthesis

14. REFERENCES

American Psychiatric Association. (2022). *Diagnostic and Statistical Manual of Mental Disorders*.

Bastien, C. H., Vallières, A., & Morin, C. M. (2001). Validation of the insomnia severity index as an outcome measure for insomnia research. *Sleep Medicine*, *2*(4), 297–307. https://doi.org/10.1016/S1389-9457(00)00065-4

Boelen, P. A., & Lancee, J. (2013). Sleep difficulties are correlated with emotional problems following loss and residual symptoms of effective prolonged grief disorder treatment. *Depression Research and Treatment*, *2013*, 1–6. https://doi.org/10.1155/2013/739804

Bouwmeester, S., & Jongerling, J. (2020). Power of a randomization test in a single case multiple baseline AB design. *PLoS ONE*, *15*(2), 1–21. https://doi.org/10.1371/journal.pone.0228355

Brown, L. F., Reynolds, C. F., Monk, T. H., Prigerson, H. G., Dew, M. A., Houcka, P. R., Mazumdar, S., Buysse, D. J., Hoch, C. C., & Kupfer, D. J. (1996). Social rhythm stability following late-life spousal bereavement: Associations with depression and sleep impairment. *Psychiatry Research*, *62*(2), 161–169. https://doi.org/10.1016/0165-1781(96)02914-9

Centraal Bureau voor de Statistiek. (2024). *Sterfte* [Webpagina]. Retrieved June 26, 2024, from https://www.cbs.nl/nl-nl/visualisaties/dashboard-bevolking/bevolkingsgroei/overlijden

Christensen, H., Batterham, P. J., Gosling, J. A., Ritterband, L. M., Griffiths, K. M., Thorndike, F. P., Glozier, N., O'Dea, B., Hickie, I. B., & Mackinnon, A. J. (2016). Effectiveness of an online insomnia program (SHUTi) for prevention of depressive episodes (the GoodNight Study): A randomised controlled trial. *The Lancet Psychiatry*, *3*(4), 333–341. https://doi.org/10.1016/S2215-0366(15)00536-2

de Lang, T. A., Buyukcan-Tetik, A., de Jong, P. J., Lancel, M., & Eisma, M. C. (2023). Cross-Lagged Analyses of Prolonged Grief and Depression Symptoms With Insomnia

Symptoms. *Behavior Therapy*, *54*(3), 510–523. https://doi.org/10.1016/j.beth.2022.12.004

de Lang, T. A., Buyukcan-Tetik, A., de Jong, P. J., Lancel, M., & Eisma, M. C. (2024). Trajectories of insomnia following bereavement. *Sleep Medicine*, *114*(August 2023), 159–166. https://doi.org/10.1016/j.sleep.2023.12.009

Doering, B. K., & Eisma, M. C. (2016). Treatment for complicated grief. *Current Opinion in Psychiatry*, *29*(5), 286–291. https://doi.org/10.1097/YCO.0000000000000263

Freeman, D., Sheaves, B., Waite, F., Harvey, A. G., & Harrison, P. J. (2020). Sleep disturbance and psychiatric disorders. *The Lancet Psychiatry*, *7*(7), 628–637. https://doi.org/10.1016/S2215-0366(20)30136-X

Gee, B., Orchard, F., Clarke, E., Joy, A., Clarke, T., & Reynolds, S. (2019). The effect of non-pharmacological sleep interventions on depression symptoms: A meta-analysis of randomised controlled trials. *Sleep Medicine Reviews*, *43*, 118–128. https://doi.org/10.1016/j.smrv.2018.09.004

Hertenstein, E., Benz, F., Schneider, C. L., & Baglioni, C. (2023). Insomnia—A risk factor for mental disorders. *Journal of Sleep Research*, *32*(6). https://doi.org/10.1111/jsr.13930

Ho, F. Y. Y., Chan, C. S., & Tang, K. N. S. (2016). Cognitive-behavioral therapy for sleep disturbances in treating posttraumatic stress disorder symptoms: A meta-analysis of randomized controlled trials. *Clinical Psychology Review*, *43*, 90–102. https://doi.org/10.1016/j.cpr.2015.09.005

Irwin, M. R., Carrillo, C., Sadeghi, N., Bjurstrom, M. F., Breen, E. C., & Olmstead, R. (2022). Prevention of Incident and Recurrent Major Depression in Older Adults With Insomnia. *JAMA Psychiatry*, *79*(1), 33. https://doi.org/10.1001/jamapsychiatry.2021.3422

Johannsen, M., Damholdt, M. F., Zachariae, R., Lundorff, M., Farver-Vestergaard, I., & O’Connor, M. (2019). Psychological interventions for grief in adults: A systematic review and meta-analysis of randomized controlled trials. *Journal of Affective Disorders*, *253*, 69–86. https://doi.org/10.1016/j.jad.2019.04.065

Kazdin, A. E. (2019). Single-case experimental designs. Evaluating interventions in research and clinical practice. *Behaviour Research and Therapy*, *117*(April 2018), 3–17. https://doi.org/10.1016/j.brat.2018.11.015

Kerkhof, G. A., Geuke, M. E. H., Brouwer, A., Rijsman, R. M., Schimsheimer, R. J., & Van Kasteel, V. (2013). Holland Sleep Disorders Questionnaire: A new sleep disorders questionnaire based on the International Classification of Sleep Disorders-2. *Journal of Sleep Research*, *22*(1), 104–107. https://doi.org/10.1111/j.1365-2869.2012.01041.x

Komischke-Konnerup, K. B., Zachariae, R., Boelen, P. A., Marello, M. M., & O'Connor, M. (2024). Grief-focused cognitive behavioral therapies for prolonged grief symptoms: A systematic review and meta-analysis. *Journal of Consulting and Clinical Psychology*, *92*(4), 236–248. https://doi.org/10.1037/ccp0000884Kyle, S. D., Miller, C. B., Rogers, Z., Siriwardena, A. N., MacMahon, K. M., & Espie, C. A. (2014). Sleep Restriction Therapy for Insomnia is Associated with Reduced Objective Total Sleep Time, Increased Daytime Somnolence, and Objectively Impaired Vigilance: Implications for the Clinical Management of Insomnia Disorder. *Sleep*, *37*(2), 229–237. https://doi.org/10.5665/sleep.3386

Lancee, J., Van Straten, A., Morina, N., Kaldo, V., & Kamphuis, J. H. (2016). Guided online or face-to-face cognitive behavioral treatment for insomnia: A randomized wait-list controlled trial. *Sleep*, *39*(1), 183–191. https://doi.org/10.5665/sleep.5344

Lancel, M., Stroebe, M., & Eisma, M. C. (2020). Sleep disturbances in bereavement: A systematic review. *Sleep Medicine Reviews*, *53*, 101331. https://doi.org/10.1016/j.smrv.2020.101331

Lenferink, L. I. M., Eisma, M. C., Smid, G. E., de Keijser, J., & Boelen, P. A. (2022). Valid measurement of DSM-5 persistent complex bereavement disorder and DSM-5-TR and ICD-11 prolonged grief disorder: The Traumatic Grief Inventory-Self Report Plus

(TGI-SR+). *Comprehensive Psychiatry*, *112*, 152281. https://doi.org/10.1016/j.comppsych.2021.152281

Lenferink, L. I. M., Franzen, M., ten Klooster, P. M., Knaevelsrud, C., Boelen, P. A., & Heeke, C. (2023). The Traumatic Grief Inventory-Clinician Administered: A psychometric evaluation of a new interview for ICD-11 and DSM-5-TR prolonged grief disorder severity and probable caseness. *Journal of Affective Disorders*, *330*(March), 188–197. https://doi.org/10.1016/j.jad.2023.03.006

Lundorff, M., Holmgren, H., Zachariae, R., Farver-Vestergaard, I., & O'Connor, M. (2017). Prevalence of prolonged grief disorder in adult bereavement: A systematic review and meta-analysis. *Journal of Affective Disorders*, *212*, 138–149. https://doi.org/10.1016/J.JAD.2017.01.030

Maric, M., & van der Werff, V. (2020). Single-Case Experimental Designs in Clinical Intervention Research. In *Small Sample Size Solutions* (pp. 102–111). Routledge. https://doi.org/10.4324/9780429273872-9

Michiels, B., & Onghena, P. (2019). Randomized single-case AB phase designs: Prospects and pitfalls. *Behavior Research Methods*, *51*(6), 2454–2476. https://doi.org/10.3758/s13428-018-1084-x

Mijnster, T., Boersma, G. J., Meijer, E., & Lancel, M. (2022). Effectivity of (Personalized) Cognitive Behavioral Therapy for Insomnia in Mental Health Populations and the Elderly: An Overview. *Journal of Personalized Medicine*, *12*(7), 1070. https://doi.org/10.3390/jpm12071070

Monk, T. H., Germain, A., & Reynolds, C. F. (2008). Sleep disturbance in bereavement. *Psychiatric Annals*, *38*(10), 671–675. https://doi.org/10.3928/00485713-20081001-06

Morin, C. M. (1993). *Insomnia: Psychological assessment and management.* The Guilford Press.

Morin, C. M., Jarrin, D. C., Ivers, H., Mérette, C., Leblanc, M., & Savard, J. (2020). Incidence, Persistence, and Remission Rates of Insomnia over 5 Years. *JAMA Network Open*, *3*(11), 1–11. https://doi.org/10.1001/jamanetworkopen.2020.18782

Morley, S. (2018). *Single-Case Methods in Clinical Psychology A Practical Guide*.

Parker, R. I., Vannest, K. J., Davis, J. L., & Sauber, S. B. (2011). Combining Nonoverlap and Trend for Single-Case Research: Tau-U. *Behavior Therapy*, *42*(2), 284–299. https://doi.org/10.1016/j.beth.2010.08.006

Qaseem, A., Kansagara, D., Forciea, M. A., Cooke, M., Denberg, T. D., Barry, M. J., Boyd, C., Chow, R. D., Fitterman, N., Harris, R. P., Humphrey, L. L., Manaker, S., McLean, R., Mir, T. P., Schünemann, H. J., Vijan, S., & Wilt, T. (2016). Management of chronic insomnia disorder in adults: A clinical practice guideline from the American college of physicians. *Annals of Internal Medicine*, *165*(2), 125–133. https://doi.org/10.7326/M15-2175

Riemann, D., Espie, C. A., Altena, E., Arnardottir, E. S., Baglioni, C., Bassetti, C. L. A., Bastien, C., Berzina, N., Bjorvatn, B., Dikeos, D., Dolenc Groselj, L., Ellis, J. G., Garcia-Borreguero, D., Geoffroy, P. A., Gjerstad, M., Gonçalves, M., Hertenstein, E., Hoedlmoser, K., Hion, T., … Spiegelhalder, K. (2023). The European Insomnia Guideline: An update on the diagnosis and treatment of insomnia 2023. *Journal of Sleep Research*, *32*(6), 1–36. https://doi.org/10.1111/jsr.14035

Riemann, D., Spiegelhalder, K., Nissen, C., Hirscher, V., Baglioni, C., & Feige, B. (2012). REM Sleep Instability – A New Pathway for Insomnia? *Pharmacopsychiatry*, *45*(5), 167–176. https://doi.org/10.1055/s-0031-1299721

Sveen, J., Jernelöv, S., Pohlkamp, L., Kreicbergs, U., & Kaldo, V. (2021). Feasibility and preliminary efficacy of guided internet-delivered cognitive behavioral therapy for insomnia after the loss of a child to cancer: Randomized controlled trial. *Internet Interventions*, *25*, 100409. https://doi.org/10.1016/j.invent.2021.100409

Taylor, D. J., Wilkerson, A. K., Pruiksma, K. E., Williams, J. M., Ruggero, C. J., Hale, W., Mintz, J., Organek, K. M., Nicholson, K. L., Litz, B. T., Young-McCaughan, S., Dondanville, K. A., Borah, E. V., Brundige, A., & Peterson, A. L. (2018). Reliability of the Structured Clinical Interview for DSM-5 Sleep Disorders Module. *Journal of Clinical Sleep Medicine*, *14*(03), 459–464. https://doi.org/10.5664/jcsm.7000

van der Zweerde, T., Lancee, J., Slottje, P., Bosmans, J. E., Van Someren, E. J. W., & Van Straten, A. (2020). Nurse-guided internet-delivered cognitive behavioral therapy for insomnia in general practice: Results from a pragmatic randomized clinical trial. *Psychotherapy and Psychosomatics*, *89*(3), 174–184. https://doi.org/10.1159/000505600

Vandekerckhove, M., & Wang, Y. (2018). Emotion, emotion regulation and sleep: An intimate relationship. *AIMS Neuroscience*, *1*(1), 1–22. https://doi.org/10.3934/Neuroscience.2018.1.1

Verbeek, I., & van de Laar, M. (2023). *Behandeling van langdurige slapeloosheid*. Bohn Stafleu van Loghum. https://doi.org/10.1007/978-90-368-2946-5Wagner, B., Rosenberg, N., Hofmann, L., & Maass, U. (2020). Web-Based Bereavement Care: A Systematic Review and Meta-Analysis. *Frontiers in Psychiatry*, *11*, 525. https://doi.org/10.3389/fpsyt.2020.00525
